# Dexras1 Deletion and Iron Chelation Promote Neuroprotection in Experimental Optic Neuritis

**DOI:** 10.1038/s41598-019-48087-3

**Published:** 2019-08-12

**Authors:** Reas S. Khan, Bailey Baumann, Kimberly Dine, Ying Song, Joshua L. Dunaief, Sangwon F. Kim, Kenneth S. Shindler

**Affiliations:** 1 Scheie Eye Institute, Perelman School of Medicine, University of Pennsylvania, Philadelphia, PA, 19104 USA; 2 FM Kirby Center for Molecular Ophthalmology, University of Pennsylvania, Stellar-Chance Laboratories, 3rd Floor, 422 Curie Blvd, Philadelphia, PA 19104 USA; 3 School of Veterinary Medicine, University of Pennsylvania, Philadelphia, PA 19104 USA; 4 Division of Endocrinology, Diabetes, and Metabolism, Department of Medicine, Johns Hopkins University School of Medicine, Baltimore, MD 21224 USA; 5 Department of Neuroscience, Johns Hopkins University School of Medicine, Baltimore, MD 21224 USA

**Keywords:** Neuroimmunology, Experimental models of disease, Translational research, Multiple sclerosis, Neurodegeneration

## Abstract

Dysregulation of iron metabolism, and resultant cytotoxicity, has been implicated in the pathogenesis of multiple sclerosis (MS) and other neurodegenerative processes. Iron accumulation promotes cytotoxicity through various mechanisms including oxidative stress and glutamate toxicity, and occurs in both MS patients and in the experimental autoimmune encephalomyelitis (EAE) model of MS. Divalent Metal Transporter1, a major iron importer in cells, is stimulated by signaling of Dexras1, a small G protein member of the Ras family. Dexras1 is activated by S-nitrosylation by nitric oxide (NO) produced by either inducible nitric oxide synthase in activated microglia/macrophages or neuronal nitric oxide synthase in neurons. Here we show Dexras1 exacerbates oxidative stress-induced neurodegeneration in experimental optic neuritis, an inflammatory demyelinating optic nerve condition that occurs in MS and EAE. Dexras1 deletion, as well as treatment with the iron chelator deferiprone, preserves vision and attenuates retinal ganglion cell (RGC) and axonal loss during EAE optic neuritis. These results suggest that iron entry triggered by NO-activated Dexras1 signaling is a potential mechanism of neuronal death in experimental optic neuritis. The current data suggest modulation of Dexras1 signaling and iron chelation are potential novel treatment strategies for optic neuritis and MS, and possibly other optic neuropathies as well.

## Introduction

Optic neuritis is an inflammatory demyelinating disease of the optic nerve often associated with the autoimmune central nervous system disease multiple sclerosis (MS)^1^. Acute vision loss occurring during optic neuritis secondary to inflammation and demyelination typically improves over several weeks; however, some permanent vision loss persists in 60% of patients^1^ and correlates with retinal ganglion cell (RGC) axonal loss, a prominent feature of optic neuritis^2,3^. Currently optic neuritis is frequently treated with corticosteroids, which reduce inflammation and hasten visual recovery, but show no improvement in final visual outcome due to their inability to prevent permanent neuronal damage^4,5^. Similarly, immunomodulatory therapies used to treat MS are effective at reducing the frequency of inflammatory episodes of the disease, but have less effect on neuronal damage and permanent neurological dysfunction^6,7^. Corticosteroids do not prevent optic nerve atrophy following optic neuritis^7,8^. Beta-Interferon treatment does not always slow the progression of axonal injury in MS^8^. Thus, therapies that prevent permanent neuronal damage and have potential to prevent long term visual loss are needed to fulfill unmet needs in the treatment of optic neuritis and MS.

Iron has been suggested to play a critical role in MS disease progression^9^. Abnormal iron homeostasis and deposition due to inflammation may involve blood brain barrier dysfunction and consequent disturbed microcirculation, alterations in iron usage or clearance due to axonal dysfunction, and dysregulation of iron transport proteins in brain tissue, all of which may contribute to neurodegeneration^10^. Pathological accumulation of iron within tissues leads to generation of free radicals, resulting in oxidative stress, which has been implicated in the pathogenesis of MS, potentially contributing to both demyelination and axonal damage^11^. Several studies have suggested that iron chelation, by sequestering circulating free iron and reducing free radical toxicity, could be a viable strategy to reduce spinal cord disease in EAE^12–14^, although effects on RGCs and optic nerve dysfunction were not reported, and the mechanisms by which free iron enters neurons and mediates neuronal damage in EAE are not fully understood.

Dexras1 is a small G protein activated by nitric oxide (NO), which can be produced by inducible NO synthase (iNOS) in activated microglia/macrophages or by neuronal NO synthase (nNOS) in neurons^15^, two cell types intimately involved in optic neuritis. NO-mediated S-nitrosylation activates Dexras1, promoting its binding to the ACBD3 protein, which in turn binds to divalent metal transporter 1 (DMT1), a major iron importer^16^. Our previous studies using Dexras1 knock out mice show that Dexras1 deletion prevents RGC death induced by NMDA by abolishing iron uptake and neurotoxicity^17^, but the model used represents an artificial toxic insult as opposed to a disease process. The potential role of Dexras1 signaling in disease pathogenesis remains unclear and needs further investigation. Iron redistribution is associated with pathogenesis and progression of many neurodegenerative diseases including MS^18,19^, Parkinson’s disease^20^, Alzheimer’s disease^21^, and age-related macular degeneration^22^.

While increased iron levels have been noted in MS patients, and iron chelation reduces spinal cord disease in EAE, the potential role of iron and iron uptake in optic neuritis, and specifically in RGCs, is not clear. Therefore, in the present study, we investigated whether Dexras1 deletion or iron chelation reduces RGC damage in EAE by reducing neurotoxicity.

## Materials and Methods

### Animals

Six-week-old female C57BL/6J mice were purchased from the Jackson Laboratory (Bar Harbor, ME, USA), and Dexras1 KO mice (on a C57BL/6J background)^17^ were bred at the University of Pennsylvania. Mice were housed at the animal facility at the University of Pennsylvania and were fed with a standard laboratory diet with free access to water with or without Deferiprone (DFP, 1 mg/mL) *ad libitum*. Mice were housed in a 12 h light/dark cycle at an ambient temperature of 22 °C and fed standard rodent chow. A subset of mice was treated with DFP in the drinking water, where indicated, starting on day1 post immunization until sacrifice. All procedures were approved by and conformed to Institutional Animal Care and Use Committee at the University of Pennsylvania guidelines. All applicable international, national, and institutional guidelines for the care and use of animals were followed.

### Generation and maintenance of Dexras1 knock-out mice

Methods used to generate the Dexras1 KO mouse line have been previously published^17^. Briefly, the gene encoding mouse Dexras1, *Rasd1*, is located on chromosome 17 and consists of two exons. Rasd1^+/−^ mice were generated using a targeting construct based on the sequence of the C57BL/6 strain *Rasd1* gene (GenBank accession number AF239157). The PGK-neo selection cassette was inserted downstream of exon 2. The PGK-neo cassette was flanked by flippase recognition target (FRT) sites and deleted with enhanced flippase recombinase. All the exons were flanked by loxP sites and deleted with Cre recombinase. All mice were maintained on a C57BL/6J background.

### Genotyping and reverse transcription-PCR analysis

Mice were genotyped by PCR analysis of genomic DNA from tail biopsies to assess presence or absence of *Rasd1*, as in prior studies^17^. Primer sets P1 (CGATCCGCGGCGAAGTCTAC) and P2 (GCGGTGCAAGTCGGGGCTCATCT) yield a 579 bp product from the wild-type (Rasd1^+^) allele.

### Induction and scoring of EAE

EAE was induced according to prior studies^23,24^. Briefly, 8 week old female C57BL/6J mice (N = 18) were anesthetized with isoflurane and were injected subcutaneously at two sites on the back with a total of 200 μg of myelin oligodendrocyte glycoprotein (MOG) peptide (MOG_35–55_; Genscript, Piscataway, NJ, USA) emulsified in Complete Freund’s Adjuvant (Difco, Detroit, MI, USA) containing 2.5 mg/ml mycobacterium tuberculosis (Difco). Control mice (N = 9) were injected with an equal volume of phosphate buffered saline (PBS) and Complete Freund’s Adjuvant. Each animal also received 200 ng pertussis toxin (List Biological, Campbell, CA, USA) in 0.1 ml PBS by intraperitoneal injection at 0 h and 48 h post-immunization. Severity of EAE was scored using a previously published^23–25^ 5-point scale: no disease = 0; partial tail paralysis = 0.5; tail paralysis or waddling gait = 1.0; partial tail paralysis and waddling gait = 1.5; tail paralysis and waddling gait = 2.0; partial limb paralysis = 2.5; paralysis of one limb = 3.0; paralysis of one limb and partial paralysis of another = 3.5; paralysis of two limbs = 4.0; moribund state = 4.5; death = 5.0.

### Quantification of RGC numbers

RGCs were immunolabeled with Brn3a and counted as described previously^23,24^. Briefly, mice (N = 19) were anesthetized with ketamine (100 mg/kg) and xylazine (10 mg/kg), and sacrificed by cardiac perfusion with phosphate buffered saline (PBS) and 4% paraformaldehyde (PFA) in PBS. Each eye was removed and further post-fixed in 4% PFA overnight at 4 °C. Retinas were dissected, washed twice with PBS, and then permeabilized in 0.5% Triton X-100 in PBS at −70 °C for 15 min followed by washing in PBS containing 0.5% Triton X-100. Retinas were then incubated overnight at 4 °C with goat-anti-Brn3a (RGC marker) primary antibody (Santa Cruz Biotechnology, Dallas, TX, USA) diluted 1:100 in blocking buffer made of PBS containing 2% bovine serum albumin and 2% Triton X-100. Following 3 washes in PBS, specimens were incubated with Alexa Fluor 488-conjugated anti-goat secondary antibody (Thermo Fisher Scientific, Waltham, MA, USA), diluted 1: 500 in blocking buffer for 2 hours at room temperature. After 3 to 4 washes in PBS, specimens were mounted vitreous side up on slides in fluorescent anti-fading solution. RGCs were photographed at 20× magnification in 12 standard fields: 1/6, 3/6, and 5/6 of the retinal radius from the center of the retina in each quadrant, and counted by a masked investigator using image analysis software (Image-Pro Plus 5.0; Media Cybernetics, Silver Spring, MD).

### Measurement of optokinetic responses (OKR)

Optokinetic responses (OKR) were used to evaluate visual function in control (N = 5) and wild type EAE mice (N = 5), Dexras1 KO EAE mice (N = 5), and wild type EAE mice treated with DFP (N = 4). OKR function is determined by the highest spatial frequency at which mice track a 100% contrast grating projected at varying spatial frequencies using OptoMetry software and apparatus (Cerebral Mechanics Inc., Lethbridge, AB, Canada), as in prior studies^23–25^. Data are reported as cyc/deg.

### Quantification of RGC axon staining

The density of intact RGC axons was quantified using anti-neurofilament immunostaining in optic nerve sections according to a previously published protocol^23,25^. Briefly, optic nerves were isolated from the same mice used for RGC quantification, and were washed and embedded in paraffin. 5 μm longitudinal paraffin sections were deparaffinized, rehydrated, and then permeabilized with 0.5% tween-20 in PBS. Nonspecific binding was reduced using Blocking reagent (Vector Laboratories, Burlingame, CA, USA). Specimens were then incubated in rabbit anti-neurofilament antibody 1: 100 (Abcam, Cambridge, MA, USA) at 4 °C overnight, washed in PBS, then incubated with anti-rabbit secondary antibody (Vectastain Elite ABC Rabbit kit) for 30 min at 37 °C. Avidin/Biotin Complex detection was performed using the Vectastain Elite ABC kit and DAB (diaminobenzidine) substrate kit (Vector Laboratories) according to the manufacturer’s instructions. Photographs of three fields/nerve (one each at the distal, central, and proximal regions of the longitudinal optic nerve section) at 20× magnification were taken by a masked investigator. Neurofilament staining was quantified by calculating the optical density using ImageJ software (http://nih.gov).

### Quantification of inflammation and demyelination in the optic nerve

Optic nerve sections were stained with Hematoxylin and Eosin (H&E) to assess inflammation and Luxol fast blue (LFB) to assess demyelination as in prior studies^23,25^. The entire length of each optic nerve section was examined by light microscopy by a masked investigator. Presence of inflammatory cell infiltration in H&E stained optic nerves was scored on a 0–4 point scale: 0 = no infiltration; 1 = mild cellular infiltration of the optic nerve or optic nerve sheath; 2 = moderate infiltration; 3 = severe infiltration; and 4 = massive infiltration. Demyelination was scored on a 0–3 point scale by examination of the entire length of LFB-stained optic nerve sections: 0 = no demyelination; 1 = scattered foci of demyelination; 2 = prominent foci of demyelination; and 3 = large (confluent) areas of demyelination. To further quantify inflammatory cell infiltration by macrophages/microglia, optic nerves were immunostained using anti-Iba1 antibodies according to prior studies^23^. Briefly, antigen retrieval was done by heating deparaffinized optic nerve sections at 95 °C in Vector antigen unmasking solution (Vector Laboratories, Burlingame, CA, USA) for 15 minutes. Specimens were incubated for 1 hr in blocking reagent from Vectastain Elite Avidin/Biotin Complex kit (ABC; Vector Laboratories), and sections were then incubated in rabbit anti-Iba1 antibody 1:200 (WAKO, Richmond, VA, USA) at 4 °C overnight. Sections were incubated with goat biotinylated anti-rabbit secondary antibody (Invitrogen, Carlsbad, CA, USA) for 2 hours at room temperature after washing in PBS. Avidin/Biotin Complex detection was performed using the Vectastain Elite ABC kit and DAB substrate kit (Vector Laboratories) according to the manufacturer’s instructions. Three standardized photos were taken at the distal, central, and proximal regions of each optic nerve, and the total number of Iba1+ cells was counted in each nerve by a masked investigator.

### Primary pan retinal and purified RGC cultures

Primary pan retinal cells were isolated according to prior studies^23,26^. Briefly, retina was removed from 4 to 5 day old C57BL/6J mice immediately following euthanization by cervical dislocation. Cells were dissociated in solution containing 0.45 U papain (Worthington, Lakewood, NJ) for 30 min at 37 °C. Papain was deactivated with ovomucoid and tissues were triturated in neurobasal medium containing SATO supplement, NS21 supplement and growth factors- brain-derived neurotrophic factor (BDNF), ciliary neurotrophic factor (CNTF), and Forskolin, and then passed through a 40 μM cell strainer. Cells were counted and then seeded onto poly-D-lysine (0.1 mg/ml, molecular weight <300,000 Da, Sigma) and laminin (20 μg/ml, Sigma) coated 96 well plates at a cell density of 1 × 10^5^ cells per mL. Alternatively, triturated retinal cells were subjected to a two-step immunopanning method for RGC purification based on a previously published protocol^27^. Briefly, 10 cm culture dishes were coated with 10 µg/ml affinity-purified goat anti-mouse IgG (H + L) antibody (Jackson Immunology Lab, West Grove, PA) in 50 mM Tris (pH 9.5) at 4 °C overnight. The dishes were then incubated with 2 µg/ml of mouse anti-mouse Thy1.2 antibody (Biorad, Hercules, CA) for 2 hr at room temperature. The plates were then washed several times, stored at 4 °C, and used within 2 days. Two negative selection plates were made by adding 5 µG/mL of Lectin from Bandeiraea simplicifolia (BSL-1, Vector Labs, Burlingame, CA) in sterile DPBS for 2 hr and then washed with PBS. The retinal cell suspension was incubated in BSL-1 plate for 45 min with agitation every 15 min to ensure good cell adhesion. Non-adherent cells were transferred to the second dish pre-coated BSL-1 and incubated for another 30 min. The non-adherent cells were then put in the dish pre-coated with mouse anti-mouse Thy1.2 antibody and incubated for 1 h with agitation every 15 min. After washing several times with DPBS, adherent cells were then detached by trypsinization using 0.05% trypsin-EDTA (Life Technologies) in PBS for 5 min at 37 °C. The cells were resuspended in Neurobasal medium containing SATO supplement, NS21 supplement and growth factors and plated at a density 5,000 cells/cm^2^ on a pre-coated 96 well plate with poly-L-lysine and laminin.

### Iron uptake study

Iron uptake assays were performed as previously described^16^. The cells were washed with PBS then resuspended into Iron Uptake Buffer (25 mM Tris, 25 mM MES, 140 mM NaCl, 5.4 mM KCl, 5 mM glucose, 1.8 mM CaCl_2_ [pH 5.5]) and transferred to glass test tubes. Ascorbic acid was added to 1 mM FeSO_4_ at a 44:1 ratio. ^55^FeCl_3_ (PerkinElmer Life Science) was added to the iron/ascorbic acid mixture, which was then added to the cells in Iron Uptake Buffer to a final concentration of 20 μM. Cells were incubated at 37 °C with shaking for 30 min. The cells were washed twice with cold PBS plus 0.5 mM EDTA and harvested. An aliquot of resuspended cells was taken for protein assay using the Bio-Rad Protein Assay Reagent; the protein concentrations of individual samples were used to quantitate ^55^Fe incorporation (cpm/μg protein). Samples were normalized to control.

### Iron imaging assay

Cells were loaded with 20 μM Phen Green SK (Invitrogen) in culture medium at 37 °C for 20 min followed by a 15 min wash. Cellular fluorescence was excited at 488 nm and quenching of fluorescence was induced by the addition of the membrane-permeable transition metal chelator 2,2′-bipyridyl (5 mM). The 2,2′-bipyridyl analogue 4,4′-bipyridyl, which cannot bind or chelate Fe^2+^, was used as a control. Because many variables such as dye loading may contribute to the variation of basal fluorescence (*F*) of PG SK, the normalized change of fluorescence (Δ*F*/*F*) was used as readout to estimate the change in cytosolic Fe^2+^ levels.

### Cell viability assay

The cell viability was determined by 3-(4,5-demerthylthiazol-2-yl)-2, 5-diphenyltetrazolium bromide (MTT) using a Cell Proliferation Kit (Roche, Mannheim, Germany) according to manufacturer’s instructions. Briefly, cells were seeded at a density of 2 × 10^5^ cells per well in 96-well culture plates. After 24 hr, the cells were treated with DETA NONOate (200 µM) with or without Deferiprone (200 µM) for 24 hr. After adding 10 µL of MTT reagent to each well, the plate was incubated at 37 °C for 4 hr followed by removal of the media and addition of 100 µL DMSO. The cell viability in each well was measured as the optical density at a wavelength of 570 nm using a Tecan infinite M200 pro microplate reader (Tecan, Mannedorf, Switzerland).

### Western blot analysis and biotin switch assay

Cells were homogenized by 26G needle in HEN (250 mM Hepes-NaOH pH 7.7, 1 mM EDTA, 0.1 mM Neocuproine) buffer and then centrifuged at 1000 g for 10 min at 4 °C. Cells lysates (240 µg) was added to 4 vol of blocking buffer [9 vol of HEN buffer plus 1 vol 25% SDS, adjusted to 20 mM MMTS with a 2 M stock prepared in dimethylformamide (DMF)] at 50 °C for 20 min with frequent vortexing. The MMTS was then removed by desalting three times with the MicroBioSpin6 column (Bio-Rad, Hercules, CA) preequilibrated in HEN buffer. To the eluate was added biotin–HPDP prepared fresh as a 4 mM stock in DMSO from a 50 mM stock suspension in DMF. Sodium ascorbate was added to a final concentration of 1 mM. After incubation for 1 hour at 25 °C, biotinylated proteins were precipitated by streptavidin-agarose beads. The streptavidin-agarose was then pelleted and washed 5 times with HENS buffer. The biotinylated proteins were eluted by SDS-PAGE sample buffer and subjected to Western blot analysis^28^.

### Experimental design and statistical analysis

Mice were randomly numbered and assigned to treatment groups, and all vision testing and tissue analysis was performed by masked investigators. Data are expressed as means ± standard error of the mean (SEM). Differences in OKR across time were compared by analysis of variance (ANOVA) of repeated measures using GraphPad Prism 5.0 (GraphPad Software, San Diego, CA, USA). Differences in RGC numbers, RGC axon staining, inflammation, and demyelination were compared using one-way ANOVA followed by Student-Newman-Keuls post-hoc analysis using GraphPad Prism 5.0. Differences in iron marker expression between wild-type and EAE mice were compared by Student’s t-test using GraphPad Prism 5.0. Differences were considered statistically significant at *p* < 0.05 for all comparisons. Retinas isolated from the right eye of each mouse were used to label and count RGCs. Because prior studies have shown that optic neuritis can occur bilaterally, or unilaterally in either eye, and thus occurs as an independent event, OKR data were first analyzed considering each eye as an independent data point similar to prior studies^23–25^. OKR was also re-analyzed using data from right eyes only to confirm that statistical trends were unaffected by within-subject inter-eye correlations. For subsequent measurements of optic nerve axon loss, demyelination and inflammation, each eye was used as an independent data point similar to prior studies^23–25^.

## Results

### EAE induction upregulates the Dexras1 signaling pathway

**Figure 1:**
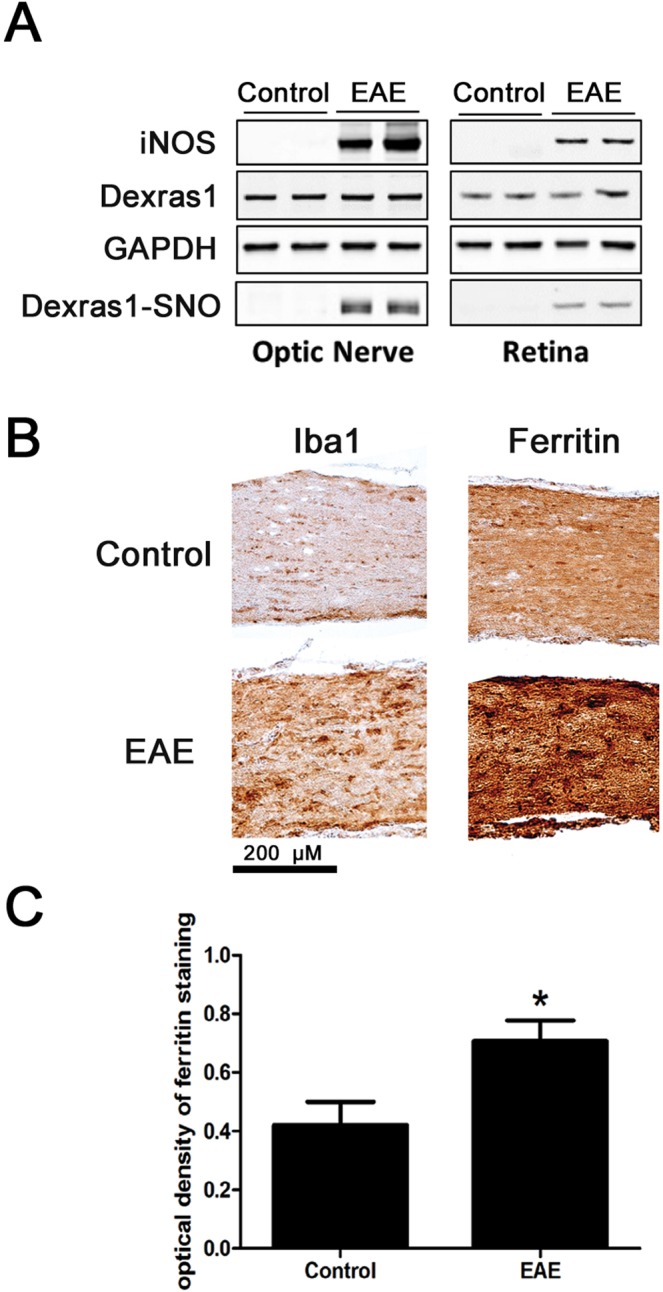
EAE induction upregulates proteins involved in iron uptake through the Dexras1 signaling pathway. Protein expression levels for iNOS, Dexras1 and GAPDH were measured by immunoblotting in control (N = 4 mice) and EAE (N = 4) mice 15 days post immunization and S-nitrosylation of Dexras1 (Dexras1-SNO) was analyzed by biotin switch assay. (**A**) Dexras1 is constitutively expressed in optic nerve and retina and becomes nitrosylated by day 15 after EAE induction (representative Western blot of proteins from optic nerves and retinas of two control and two EAE mice is shown). iNOS, not normally found in optic nerve or retina, is upregulated after EAE induction. (**B**) Control and EAE mouse optic nerves were immunolabeled with ferritin or Iba1 antibodies. EAE optic nerves show increased ferritin and Iba1, consistent with increased iron levels and an increase in Iba1+ macrophages/microglia respectively throughout the optic nerve. A representative optic nerve is shown at 20X original magnification from one control and one EAE mouse. (**C**) Optical density quantification of ferritin staining of the right optic nerves from 4 control and 4 EAE mice shows EAE optic nerves (N = 4) contain a significant (*p < 0.05) increase compared to control optic nerves (N = 4). One of three representative experiments is shown.

To evaluate whether EAE induction has a direct effect on proteins involved in Dexras1 signaling, one of the pathways that induces iron influx into the cells, proteins isolated from optic nerves and retina from control (non-EAE) and EAE mice (N = 4 mice/group) were subjected to Western blot analysis and probed for detection of iNOS, Dexras1, S-nitrosylated Dexras1 (Dexras1-SNO), and GAPDH as a housekeeping marker. Results show that non-nitrosylated Dexras1 is constitutively expressed in optic nerve and retina, but becomes nitrosylated by day 15 after EAE induction. iNOS, which is not normally expressed in optic nerves or retina, is upregulated 15 days after EAE induction (Fig. 1A). To evaluate whether EAE induces increased iron levels in the setting of optic nerve inflammation, optic nerves from control and EAE mice were immunolabeled with ferritin and Iba1 antibodies, as iron and macrophage/microglia markers, respectively. EAE optic nerves showed a significant increase (p < 0.05) in ferritin levels, suggestive of increased iron levels, and an increase in Iba1+ macrophages/microglia throughout the optic nerves confirmed the induction of optic neuritis (Fig. 1B,C).

### NO treatment induces iron uptake in retinal cultures and purified RGCs

**Figure 2:**
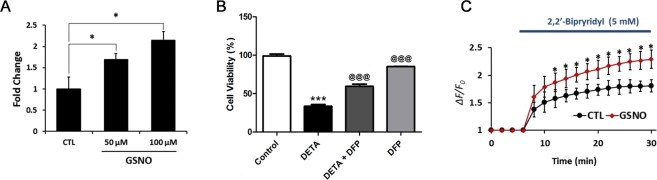
NO treatment induces iron uptake in retinal cultures and purified RGCs. Dissociated primary pan-retinal (**A**,**B**) and immunopurified RGC (**C**) cultures were prepared from neonatal day 4–5 C57BL/6J mice. (**A**) 1 hr incubation with the NO-donor GSNO induces iron uptake, measured as the presence of ascorbate-^55^Fe^2+^ (*p < 0.05). (**B**) 24 h treatment with NO-donor DETA-NONOate (200 μM) increases retinal cell death (***p < 0.001) compared with control cultures. Treatment with 200 µM deferiprone for 24 h significantly (^@@@^p < 0.001 compared with DETA-NONOate treatment alone) attenuates retinal cell death induced by DETA-NONOate. Deferiprone treatment alone (without DETA-NONOate) does not affect retinal cell survival, with no difference in cell viability compared to control cultures, and viability remains significantly higher than in cultures treated with DETA-NONOate alone (^@@@^p < 0.001). (**C**) Purified RGCs were incubated with GSNO, then intracellular iron levels were detected with iron-sensitive dye, Phen-GreenSK (PG SK) and 2,2′-bipyridyl (BPD). 2,2′-BPD chelates intracellular iron, increasing PG SK fluorescence. RGCs treated with GSNO show increased intracellular iron levels compared with control RGCs (*p < 0.05, N = 20 cells/treatment group). The normalized change of fluorescence (Δ*F*/*F*) was used to estimate change in cytosolic Fe^2+^ levels. All experiments were repeated 3 times.

To evaluate whether NO production can result in iron uptake in retinal cells, primary retinal cells were isolated from neonatal day 4–5 mouse pups. Retinal cultures were treated with NO donor S-nitrosoglutathione (GSNO) or glutathione (GSH) as control for 1 hr followed by iron uptake estimation. GSNO caused a significant (p < 0.05) dose dependent increase in iron influx into retinal cells (Fig. 2A). To evaluate toxicity induced by the influx of iron, cell viability in a pan-retinal cell culture was assessed after treatment with DETA-NONOate, which mimics long term NO release by iNOS. 24 hr treatment with DETA-NONOate showed a significant (p < 0.001) reduction in cell viability, and identical cells that were also treated with the iron chelator deferiprone (200 µM) for 24 hr showed a significant (p < 0.001) attenuation of DETANONOate-induced cell death (Fig. 2B). To determine whether NO treatment triggers iron influx specifically into RGCs, RGCs were enriched from primary retinal preparations, and immunolabeling with Brn3a confirmed over 95% purity (data not shown). Single cell iron imaging was done in purified RGCs using the iron sensitive dye PhenGreen-SK^29^, which is insensitive to major intracellular ions including physiological levels of calcium. RGCs were treated with 100 µM GSNO or GSH for 1 hr, incubated with 10 µM ascorbate Fe^2+^ for 15 minutes and then loaded with 20 µM Phen-GreenSK dye. Membrane permeable Fe specific chelator 2,2′-bipyridyl (5 mM), an indicator of iron specific fluorescent signal, was added to induce de-quenching of fluorescence. GSNO treatment displayed a significant (p < 0.05) increase in fluorescent signal upon addition of 2,2′-bipyridyl compared to control in all time points (Fig. 2C). Addition of 4,4′-bipyridyl did not affect any fluorescence (data not shown), compared to control cells, indicating NO treatment triggered influx of iron into RGCs.

### Dexras1 deletion and iron chelation preserve vision in EAE mice

**Figure 3:**
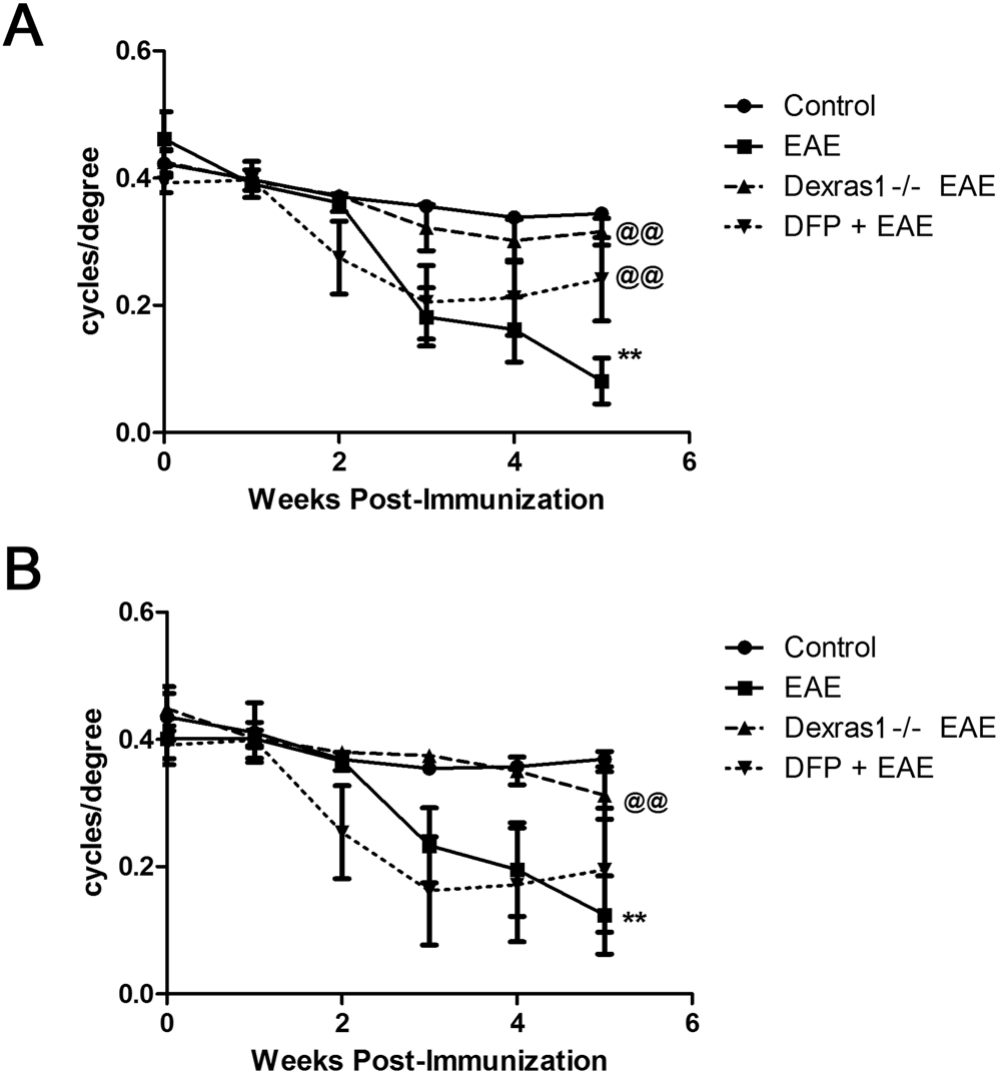
Dexras1 deletion or iron chelation preserves vision. Visual function, measured in both eyes (**A**) by weekly OKR responses, shows significant (**p < 0.01) decrease in eyes of EAE mice (N = 10 eyes of 5 mice) compared to control mouse eyes (N = 10 eyes of 5 mice) by 5 weeks after induction of EAE. Dexras1 KO mice (N = 10 eyes of 5 mice) and daily treatment with iron chelator DFP (1 mg/mL, N = 8 eyes of 4 mice) from days 0 to 42 post immunization leads to significantly (^*@@*^p < 0.01 versus wild-type EAE) improved OKR responses in EAE mice. Visual function, measured by weekly OKR responses in the right eye only (**B**) from the same mice shown in A, shows significant (**p < 0.01) decrease in eyes of EAE mice (N = 5 eyes) compared to control mouse eyes (N = 5) by 5 weeks after induction of EAE. Dexras1 KO mice (N = 5 eyes) show significantly (^*@@*^p < 0.01 versus wild-type EAE) improved OKR responses and daily treatment with iron chelator DFP (N = 4 eyes) shows a trend towards increased OKR responses.

Persistent visual deficits are common in optic neuritis patients due to axonal loss in the optic nerve^30^. Results above show Dexras1 nitrosylation increases in the optic nerve and retina, and iron levels increase in optic nerves of EAE mice. *In vitro* studies confirm NO drives iron uptake in RGCs. Thus, we hypothesized that Dexras1-mediated iron import, driven by NO induced by optic nerve inflammation in EAE optic neuritis, is a potential mechanism contributing to RGC loss and reduced visual function. To examine whether Dexras1 deletion or treatment with the known iron chelator DFP preserves vision during EAE, control mice (N = 5), wild-type EAE mice (N = 5), Dexras1 knock out (KO) EAE mice (N = 5), and wild-type EAE mice treated daily with DFP (1 mg/mL in drinking water ad libitum) (N = 4) were subjected to OKR measurement weekly after immunization until sacrifice. The DFP dose used has been shown to be protective in models of retinal degeneration and is not toxic^31–33^. EAE paralysis scores were monitored daily to confirm disease induction, and showed similar disease burden induced in each group, with an average peak EAE score of 1.5 ± 0.69 in wild-type EAE mice, 1.4 ± 0.10 in Dexras1 KO EAE mice, and 1.5 ± 0.41 in DFP-treated EAE mice. While OKR responses significantly decreased in both eyes of wild-type EAE mice versus control mice (p < 0.01) by the 5^th^ week, both Dexras1 KO mice with EAE and wild-type EAE mice treated with DFP had significantly (p < 0.01) attenuated reduction in OKR scores compared to wild-type EAE mice by 5 weeks post immunization (Fig. 3A). OKR responses from right eyes only (re-analyzed to avoid within-subject inter-eye correlations) showed significant (p < 0.01) decrease in eyes of EAE mice compared to control mouse eyes by 5 weeks post immunization. Dexras1 KO mice showed significantly (p < 0.01 versus wild-type EAE) improved OKR responses and daily treatment with iron chelator DFP (1 mg/mL) showed a trend towards increased OKR responses (Fig. 3B).

### Dexras1 deletion and iron chelation preserve RGCs and their axons in EAE mice

**Figure 4:**
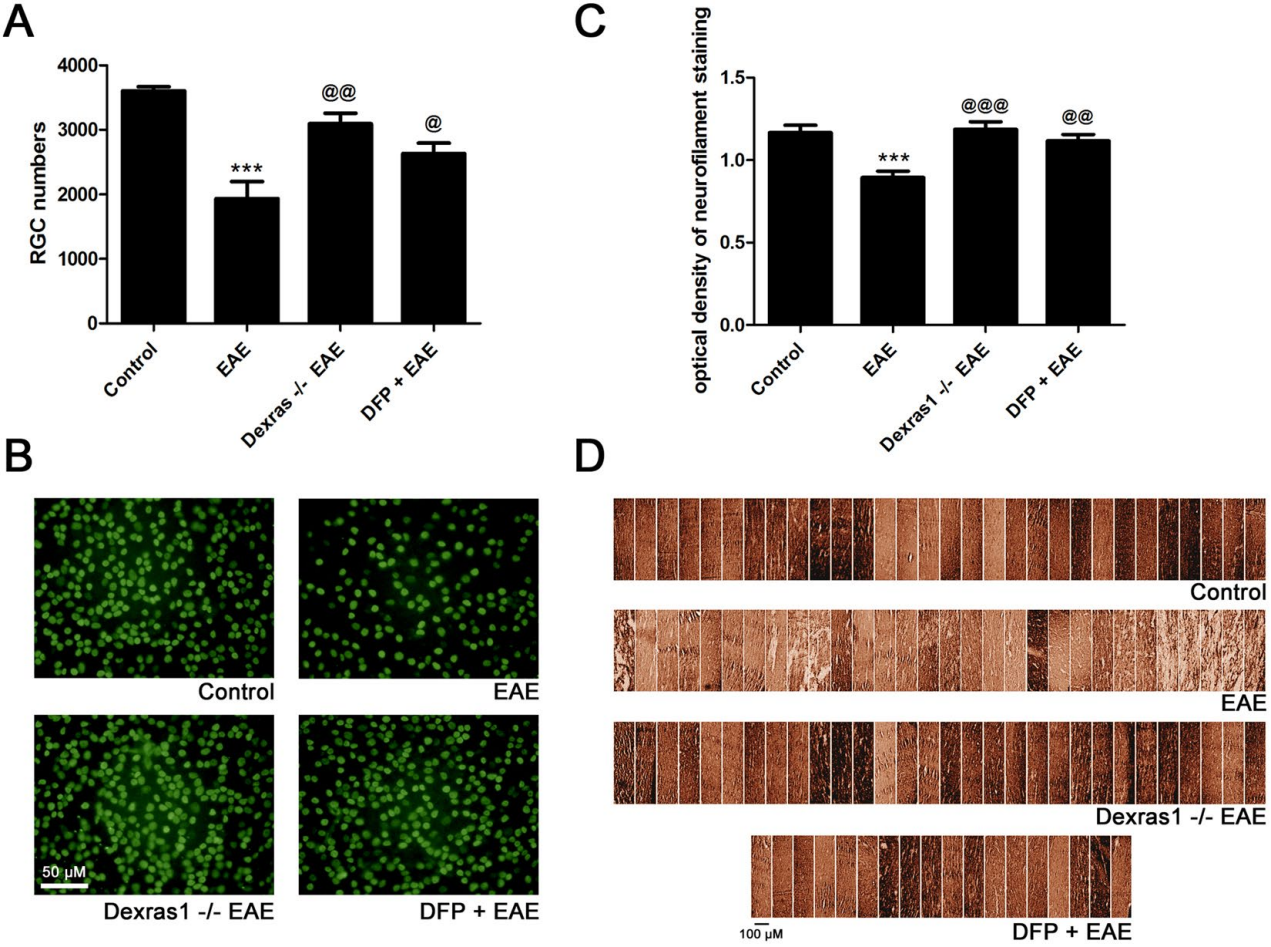
Dexras1 deletion or iron chelation preserves RGC numbers in the retina and prevents axonal loss in the optic nerves of EAE mice. Neuroprotective effects of Dexras1 deletion or iron chelation were evaluated by counting RGCs in the retina and quantifying axonal staining density in the optic nerve. RGCs were immunolabeled with Brn3a antibody and counted in a standardized, masked fashion. (**A**) RGC loss in eyes of EAE mice (***p < 0.001 versus control, N = 5 right eyes of 5 mice) is reduced in Dexras1 KO EAE mice (^@@^p < 0.01 versus EAE, N = 5 eyes of 5 mice). Daily treatment with iron chelator DFP (1 mg/mL, N = 4 eyes of 4 mice) leads to significant (^@^p < 0.05 versus EAE) improvement in RGC numbers. (**B**) Representative images show RGCs in one field of retina from each group (original magnification ×20). (**C**) Neurofilament staining was used to evaluate axonal loss in sections of optic nerves isolated at day 42 post immunization. The optical density of neurofilament staining, calculated by a masked investigator using the average of three equal-sized fields from each optic nerve, shows a significant decrease (***p < 0.001) in optic nerves (N = 10 nerves) from EAE mice compared to optic nerves (N = 10 nerves) from control mice. Dexras1 KO EAE mice (^@@@^p < 0.001, N = 10 nerves) or DFP treated EAE mice (^@@^p < 0.01, N = 6 nerves) showed a significant increase in neurofilament staining compared to optic nerves from wild type EAE mice. (**D**) A series of photographs of axon staining in three equal-sized fields from each optic nerve (one each at the distal, central, and proximal regions of the longitudinal optic nerve section) shows the normal degree of variability of neurofilament staining in optic nerves of control mice. Similar staining is seen in Dexras1 KO EAE mice and EAE mice treated with DFP, whereas optic nerves from wild-type EAE mice show more patchy loss of neurofilament staining.

Prior studies have demonstrated that in EAE, significant apoptotic RGC loss occurs, and therapies that prevent RGC loss are potentially effective in preserving vision^23–25,34,35^. To examine whether effects of Dexras1 deletion and iron chelation on visual function during EAE are in part due to prevention of RGC loss and axonal degeneration, retinas were isolated from the right eyes of the control, wild-type EAE, Dexras1 KO EAE, and wild-type EAE treated with DFP (1 mg/mL in drinking water ad libitum) mice used for OKR testing in Fig. 3, following their sacrifice on day 42 post-immunization. RGCs were counted following Brn3a staining. RGC numbers in the eyes from wild-type EAE mice showed a significant (p < 0.001) reduction compared to control mouse eyes. Dexras1 KO EAE mice (p < 0.01) and wild-type EAE mice treated with daily DFP showed significantly (p < 0.05) attenuated RGC loss compared to wild-type EAE mice (Fig. 4A,B). RGC axonal loss was assessed by neurofilament staining of optic nerve sections. Wild-type EAE mice showed significant (p < 0.001) axonal loss in the optic nerve compared to control mice, consistent with prior studies^23,25^. Dexras1 KO EAE mice (p < 0.001) and EAE mice treated with daily DFP (p < 0.01) showed a significant preservation of axons compared to wild-type EAE mice (Fig. 4C,D).

### Dexras1 deletion and DFP have limited effects on optic nerve inflammation and demyelination

**Figure 5:**
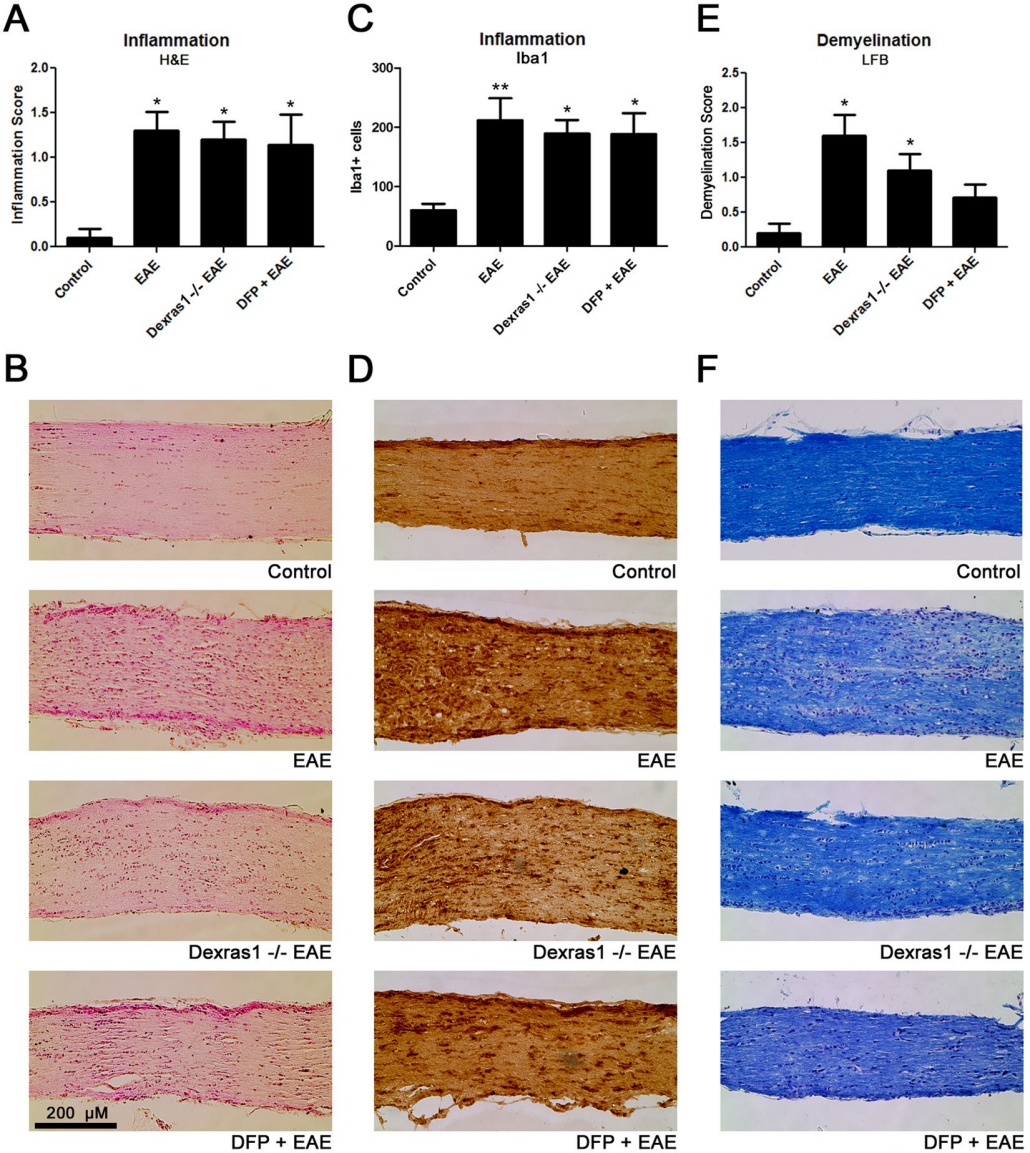
Dexras1 deletion or DFP treatment have no effect on optic nerve inflammation and just a trend towards effects on demyelination. To examine whether Dexras1 deletion or iron chelation prevents inflammation or demyelination, optic nerves were isolated from mice 42 days post immunization. (**A**) Inflammation quantified on a relative scale in H&E-stained optic nerve longitudinal sections shows optic nerves (N = 10 nerves) from EAE mice had significantly (*p < 0.05) higher inflammation scores compared to optic nerves (N = 10) from control mice. Optic nerves from Dexras1−/− EAE mice (N = 10 nerves) or DFP-treated EAE mice (N = 7 nerves) show no change in inflammation score compared to wild-type EAE mice. (**B**) A representative image of one optic nerve from a control, wild-type EAE, Dexras1−/− EAE and DFP-treated EAE mouse. Sections show increased numbers of cells, representative of inflammatory cell infiltration, except within the optic nerve from a control mouse (original magnification ×20). (**C**) To further evaluate inflammatory cell infiltration by macrophages/microglia, optic nerves were immunostained using anti-Iba1 antibodies, and the number of Iba1+ cells counted per nerve is shown. Optic nerves from EAE mice (N = 10 nerves) have more Iba1+ cells than control mouse optic nerves (N = 10, *p < 0.05), and microglia/macrophage numbers are not reduced in optic nerves from Dexras1−/− EAE (N = 10 nerves) or DFP-treated (N = 7 nerves) EAE mice. (**D**) Images show Iba1+ cells (brown) in one representative optic nerve from each group (original magnification ×20). (**E**) LFB-stained optic nerve longitudinal sections were used to quantify demyelination. Optic nerves (N = 10 nerves) from EAE mice had a significantly (*p < 0.05) higher demyelination score than optic nerves (N = 10) from control mice. Demyelination scores of Dexras1−/− EAE mice (N = 10 nerves) and DFP-treated (N = 7 nerves) EAE mice optic nerves show a trend toward less demyelination but no significant difference compared to wild-type EAE mice; however, only Dexras1−/− EAE mice (*p < 0.05) and not DFP-treated mice had increased demyelination compared with optic nerves from control mice. (**F**) A representative image of LFB staining in one optic nerve from each group (original magnification ×20) is shown.

In addition to RGC loss, optic neuritis in EAE mice involves significant inflammation and demyelination in the optic nerves^23,25,34,36^, further mechanisms that could impair visual function. To evaluate whether Dexras1 deletion or treatment with DFP induces any effect on inflammation or demyelination, optic nerves were isolated from the same mice used for OKR testing and RGC counts, following their sacrifice 42 days post immunization. Wild-type EAE mice showed a significant inflammatory cell infiltration in the optic nerve compared to control mice, measured both by H&E staining (p < 0.05) (Fig. 5A,B) and by immunolabeling macrophages/microglia with Iba1 antibodies (p < 0.01) (Fig. 5C,D). Neither Dexras1 deletion nor treatment with DFP reduced the level of inflammation detected in optic nerves of EAE mice (Fig. 5A–D). Wild-type EAE mice also showed a significant loss of myelin in the optic nerve compared to control mice (p < 0.05), measured by LFB staining (Fig. 5E,F). Dexras1 deletion did not show a statistically significant reduction in the level of demyelination in EAE mouse optic nerves, yet did exhibit a trend towards attenuation of myelin loss that was even more pronounced by treatment with DFP (Fig. 5E,F).

## Discussion

Current results demonstrate Dexras1 deletion or iron chelation provides significant neuroprotection in experimental optic neuritis by preventing visual loss, RGC loss and axonal degeneration. At the same time, Dexras1 deletion and iron chelation have no effect in preventing optic nerve inflammation, and limited or no effect on optic nerve demyelination. These results suggest that the significant improvement in visual function is mediated by promotion of RGC survival, not by an anti-inflammatory mechanism or by effects on myelin and oligodendrocytes. Thus, treatment with iron chelators or modulators of the Dexras1 signaling pathway may provide important therapeutic advantages to prevent RGC loss and related vision loss in optic neuritis, and may do so by modulating downstream factors that increase RGC loss as opposed to suppressing the optic nerve inflammatory disease itself. Iron modulation may therefore provide synergistic benefits when combined with anti-inflammatory therapies, and current results support future studies of these combined treatment strategies.

Results show that the Dexras1 pathway is upregulated in retina and optic nerve during EAE, and *in vitro* results confirm that NO, a known activator of Dexras1 signaling, induces iron uptake in RGCs and induces retinal cell death. Previous reports show that Dexras1 is a novel NO effector molecule^15^, and that Dexras1 activation via S-nitrosylation with NO leads to formation of a ternary complex that ultimately activates the DMT1 iron transporter thereby inducing iron influx into cells^16^. The effects of Dexras1 deletion or an iron chelator to prevent RGC loss in EAE mice suggest that regulation of iron may play an important role in the observed neuroprotective effects. Therefore, Dexras1 deletion or iron chelation may have potential therapeutic roles in optic neuritis that warrant further study.

Our prior studies show that immune cell infiltration occurs in the optic nerve during EAE optic neuritis and can result in a significant oxidative burst^36,37^. Interestingly, expression of iNOS and NO overproduction by phagocytes such as macrophages is recognized as one of the direct consequences of chronic disease processes^38,39^, and a major source of NO produced during oxidative burst comes from iNOS^40^. Our data show that iNOS expression is induced and Dexras1 is S-nitrosylated in both optic nerve and retina in EAE mice by day 15, a time by which inflammation has reached a peak^37^. Thus, taken together with the current results, evidence suggests that macrophage derived NO generated by iNOS may be one of the mechanisms exacerbating neuronal loss and axon degeneration during optic neuritis. Interestingly, the polyphenolic compound resveratrol has been shown previously to promote neuroprotection of RGCs by reducing oxidative stress in optic neuritis^35,36,41^, and has also been reported to inhibit iNOS expression^42^, suggesting that its neuroprotective effects in optic neuritis may be mediated at least in part by inhibition of iNOS leading to reduced iron accumulation.

Oxidative stress-mediated RGC loss is a common feature in many optic neuropathies, and also plays a key role in the pathogenesis of MS and optic neuritis^11,43^. Excessive iron in the cell is a toxic entity because of its propensity to increase the concentrations of reactive oxygen species. Iron catalyzes the generation of extremely reactive hydroxyl (^·^OH) radicals from H_2_O_2_ and superoxide through the Haber-Weiss reaction, causing severe damage to membranes, proteins and DNA^44^. We have previously shown that neuronal iron trafficking is modulated via Dexras1 which induces iron transport into the cells via DMT1, and deletion of Dexras1 abolishes chemically induced neurotoxicity in neuronal cultures and in non-physiologic conditions^17^. The current results are consistent with these prior studies, and importantly, demonstrate a significant effect on RGC survival and visual function in a disease model.

The current results showing Dexras1 deletion mediates neuroprotection in a pathological condition underscores its therapeutic implications and provides support for further studies of the role of this pathway in exacerbating RGC loss during MS and optic neuritis. Importantly, modulating iron import pathways only reduced neurodegenerative aspects of EAE optic neuritis, without suppressing the inflammatory disease mechanism that leads to neuronal injury. These findings support design of future studies of combined treatment along with anti-inflammatory therapies to both suppress optic nerve inflammation and prevent neuronal cell death. Results suggest developing potentially selective and safe Dexras1 inhibitors, in addition to broader iron chelation, holds important therapeutic potential worthy of investigation.

## Data Availability

All data in this study will be made available by the corresponding author upon request.
